# Molecular Mechanism of Silver Nanoparticles-Induced Human Osteoblast Cell Death: Protective Effect of Inducible Nitric Oxide Synthase Inhibitor

**DOI:** 10.1371/journal.pone.0164137

**Published:** 2016-10-07

**Authors:** Ewelina Zielinska, Cecylia Tukaj, Marek Witold Radomski, Iwona Inkielewicz-Stepniak

**Affiliations:** 1 Department of Medical Chemistry, Medical University of Gdansk, Gdansk, Poland; 2 Department of Electron Microscopy, Medical University of Gdansk, Gdansk, Poland; 3 College of Medicine, University of Saskatchewan, Saskatoon, Canada; 4 Kardio-Med Silesia, Zabrze, Poland; VIT University, INDIA

## Abstract

**Background:**

Silver nanoparticles (AgNPs) show strong antibacterial properties, making them excellent candidates to be used in orthopaedic repair and regeneration. However, there are concerns regarding the cytotoxicity of AgNPs and molecular mechanisms underlying AgNPs-induced bone cells toxicity have not been elucidated. Therefore, the aim of our study was to explore mechanisms of AgNPs-induced osteoblast cell death with particular emphasis on the role of nitric oxide (NO) generated by inducible nitric oxide synthase (iNOS).

**Methods and Result:**

Silver nanoparticles used in this study were 18.3±2.6 nm in size, uncoated, spherical, regular shape and their zeta potential was -29.1±2.4 mV as measured by transmission electron microscopy (TEM) and zetasizer. The release of silver (Ag) from AgNPs was measured in cell culture medium by atomic absorption spectroscopy (AAS). The exposure of human osteoblast cells (hFOB 1.19) to AgNPs at concentration of 30 or 60 μg/mL for 24 or 48 hours, respectively resulted in cellular uptake of AgNPs and changes in cell ultrastructure. These changes were associated with apoptosis and necrosis as shown by flow cytometry and lactate dehydrogenase (LDH) assay as well as increased levels of pro-apoptotic Bax and decreased levels of anti-apoptotic Bcl-2 mRNA and protein. Importantly, we have found that AgNPs elevated the levels of nitric oxide (NO) with concomitant upregulation of inducible nitric oxide synthase (iNOS) mRNA and protein. A significant positive correlation was observed between the concentration of AgNPs and iNOS at protein and mRNA level (r = 0.837, r = 0.721, respectively; p<0.001). Finally, preincubation of osteoblast cells with N-iminoethyl-l-lysine (L-NIL), a selective iNOS inhibitor, as well as treating cells with iNOS small interfering RNAs (siRNA) significantly attenuated AgNPs-induced apoptosis and necrosis. Moreover, we have found that AgNPs-induced cells death is not related to Ag dissolution is cell culture medium.

**Conclusion:**

These results unambiguously demonstrate that increased expression of iNOS and generation of NO as well as NO-derived reactive species is involved in AgNPs-induced osteoblast cell death. Our findings may help in development of new strategies to protect bone from AgNPs-induced cytotoxicity and increase the safety of orthopaedic tissue repair.

## Introduction

Orthopedic implant and medical devices are now used in patients to improve the quality of life and to save lives. This has been made possible by remarkable development of regenerative medicine and bioengineering over the past decades [1–3]. Despite this progress, implant infection still remains a serious medical and economic problem [4,5]. Microbes can form biofilms on orthopedic prosthesis resulting in local and systemic infection as well as increased risk of amputation, mortality and health care costs [3–6]. For example, the American health care system estimates the costs of prosthetic joint infection treatment at $1.62 billion in 2020 year [4]. Clinical experience has indicated that when biofilm is formed, bacteria become resistant to antibiotics, and that biofilms must be removed physically [4–6]. The advent of new nanomaterials may greatly facilitate the fight against antibiotic-resistant biofilms. Indeed, AgNPs, among other metal nanoparticles, have received particular attention [7–11]. It was demonstrated that AgNPs exerted a wide spectrum of antimicrobial activity, making them potential and promising candidate for use in the development of infection-resistant biomaterials [3,7,11–13]. AgNPs have been shown to be effective against both Gram-positive and Gram-negative bacteria as well as multidrug-resistant microbes [11]. Additionally, AgNPs exert synergistic antimicrobial effects with various antibiotics [12]. The multidirectional mechanism of antibacterial activity of AgNPs is most likely the reason why microbes develop resistance to these NPs at much slower rate when compared to antibiotics [13]. Importantly, AgNPs-coated materials show good cell and blood compatibility [8,10,14] and some of them have advanced now to clinical trials in orthopedic patients [3,7,9]. Indeed, there is still concern regarding the safety aspect of AgNPs such as cytotoxicity that limits their usage in orthopaedic implants [3,7]. Indeed, silver releasing implantable materials may induce bone damage through direct interaction with bone cells [7]. However, the information regarding cytotoxic concentrations found in literature is often contradictory [8,10,14–17] and the molecular mechanism of bone cells-induced cytotoxicity is still unclear. For example, Albers et al demonstrated that 50 nm AgNPs exerted antibacterial effects at concentrations 2–4 times higher than those causing deleterious effects on osteoblasts [17]. The significant impairment of cell viability was observed at concentration of 128 μg/mL AgNPs after 72 h. On the other hand, Pauksch et al suggested that a therapeutical window for the application of AgNPs in clinical practice might exist [18]. They found that AgNPs at a concentration of 10 μg/mL after 21 days of incubation induced impairment of human mesenchymal stem cells and osteoblasts cell viability, while some information from literature indicated that AgNPs exerted antibacterial effect at concentrations ≤ 10 μg/mL [19,20]. Despite all these contradictory data a general consensus has emerged that mechanisms of AgNPs-induced cytotoxicity need to be carefully investigated before advancing this treatment to general medical practice in order to protect patients from toxic effects of these nanoparticles.

AgNPs, similar to other NPs, have the ability to generate reactive oxygen and nitrogen species and oxidative damage in various cells [21,22]. Nitric oxide is a signaling molecule generated by nitric oxide synthase (NOS) that plays an important role in homeostasis [23]. However, aberrant generation or metabolism of NO increase the oxidizing stress and cellular damage brought about by oxidants such as peroxynitrite (ONOO^-^) and nitrogen dioxide (NO_2_) [21,24,25]. These reactive nitrogen species (RNS) react with tyrosine residues in protein to form 3-nitrotyrosine (NT) the indicators of nitrosative stress [24,25]. Both the regulatory and detrimental effects of NO have been associated with osteoblast metabolism [25–28]. Furthermore, NO was found to be implicated in xenobiotics-induced bone toxicity [29,30]. In human osteoblasts NO can be synthesized by all three NOS isoforms i.e. eNOS, nNOS and iNOS [25,31]. However, the expression of iNOS has been associated with bone injury and disease more frequently than other isoforms [25,31–33].

Considering all the information, the aim of our study was to examine mechanisms of AgNPs-induced cell injury in cultured human osteoblasts with special emphasis on the role of NO and iNOS. We felt that a better understanding of the interaction between AgNPs and osteoblast cells may improve the efficacy and safety of AgNPs-containing prosthetic devices. Therefore, we tested a range of concentrations of AgNPs to find the concentrations capable of exerting apoptosis and necrosis. Finally, we studied molecular mechanisms responsible for osteoblast cell death.

## Materials and Methods

### Characterization of AgNPs

15 nm AgNPs uncoated (water suspensions) were purchased from US Research Nanomaterials (Houston, TX, USA). The concentrations and time of incubation were selected based on results of preliminary experiments (S1 Fig).

Transmission Electron Microscope JEM-1200 EX II TEM (JEOL, Tokyo, Japan) and Dynamic light scattering (DLS) with a Zetasizer Nano ZS (Malvern Instruments, Malvern, UK) were used to characterized AgNPs used in the present study.

Briefly, TEM samples were prepared by placing a drop of the AgNP dispersed at a concentration of 1 mg/mL in ultrapure water (Milli-Q) onto a formvar-coated copper grid and drying at room temperature. AgNPs were directly examined by TEM operated at an accelerating voltage of 200 keV and diameters of 200 particles were measured to determine the mean size and size distribution.

The zeta potentials, polydisperity index and the aggregation profile (hydrodynamic diameter) of the AgNPs were obtained by DLS with a Zetasizer Nano ZS. AgNPs at concentrations of 30 and 60 μg/mL were prepared in serum-free (SF) culture medium and the measurements were performed within 1 hour four times at room temperature. The aggregation profile of AgNPs was also determined after 24, and 48 hours of incubation at 37°C and 5% CO_2_ (preconditioned medium).

1 mL of cell culture medium preconditioned with the highest working concentration of 60 μg/mL AgNPs, was used to determine the Ag release. Briefly, after centrifugation (90 min, 20 000 rpm, 0°C) the supernatant was acidified to a pH <2 with 65% HNO_3_, followed by digestion with 65 wt% HNO_3_ and 30 wt% HCl [34]. The Ag content in solution was determined using atomic absorption spectrophotometer (AAS, Atomic absorption 800, Perkin Elmer) with a detection limit of 5 μg/L. Triplicate readings were analyzed for each sample.

The supernatant, obtained after centrifugation of medium preconditioned with AgNPs, was also incubated with hFOB 1.19 cells for 24 or 48 h in order to determine potential cytotoxic effect of dissolved Ag (indicated as Ag released from AgNPs).

Additionally, all these measurements were performed for AgNPs prepared in SF culture medium in the presence of 500 μM L-NIL or 60 μM necrostatin-1 (Nec-1) (data not shown).

### Cells

Human fetal osteoblast cells (hFOB 1.19) were obtained from the American Type Culture Collection (ATCC number: CRL-11372; Manassas, VA, USA). The cells were maintained at 37°C in 5% CO_2_ in flask with a mixture of Dulbecco’s Modified Eagle’s Medium and Ham F12 medium (1:1 ratio) containing 10% fetal bovine serum, 100 U/mL penicillin, and 100 μg/mL streptomycin as previously described [35].

### Treatments

hFOB 1.19 cells were incubated with AgNPs at concentration of 30 and 60 μg/mL for 24 and 48 hours. The concentrations of AgNPs used in the study were based on results of preliminary concentration-response curves (S1 Fig). 1 mg/mL stock solutions of the AgNPs were prepared in SF culture medium, further dilutions were also made in cell culture medium. All solutions were prepared *ex tempore* every time just before adding to the cells.

According to manufacturer’s instructions AgNPs were shaken for 1 min before use to avoid nanoparticle aggregation. Each of the experiments consists of a control group: cells treated with SF culture medium without NPs. For some experiments L-N^6^ -(1-iminoethyl)- lysine (l-NIL), a selective iNOS inhibitor (Sigma-Aldrich, Poland) or Nec-1, a pharmacological inhibitor of necroptosis (Sigma-Aldrich, Poland), was added 1 hour prior the addition of AgNPs to the cells. The concentration of l-NIL (500 μM) and Nec-1 (60 μM) was selected based on preliminary experiments (S2 Fig and S3 Fig). Preincubation of cells with 500 μM L-NIL (in the absence of AgNPs) exerted no significant effects on investigated parameters.

### TEM analysis

hFOB 1.19 cells were cultured in T-25 cm^2^ flasks in complete medium until confirmed to be 80–90% confluent. Next, the cells were treated as indicated in section *Treatment*, and TEM analysis and observation with a JEM 1200 EXII TEM was carried out according to previously published method [35].

### Western blotting of iNOS, NT, Bax and Bcl-2

Western blotting method was used in order to study protein levels of iNOS, Bax and NT. Briefly, osteoblast cells were cultured in 10 cm Petri dishes and incubated with AgNPs or in combination with L-NIL as indicated in section *Treatments*. Next, cell culture media were removed, cells rinsed three times with phosphate buffer, detached and homogenized in protein lysis buffer in the presence of protease inhibitors (20 mM Tris (pH 7.5), 135 mM NaCl, 2 mM DTT, 2 mM EDTA, 2 mM sodium pyrophosphate, 25 mM b-glycerophosphate, 1% Triton X-100, 10% glycerol, 1 mM sodium orthovanadate, 10 mM NaF, 10 μg/ml aprotinin, 10 μg/ml leupeptin and 1 mM phenylmethylsulfonyl fluoride). The resultant homogenates were centrifuged, collected and protein concentrations were measured by Bradford method. The samples (40 μg protein per lane) were boiled for 5 minutes and separated by SDS–PAGE on polyacrylamide gel and transferred onto nitrocellulose membrane, which was blocked with 5% non-fat dry milk-PBST buffer (phosphate-buffered saline (PBS) containing 0.1% Tween-20) for 1 hour at room temperature and incubated at 4°C for overnight with rabbit polyclonal antibody: anti-Bax, anti-Bcl-2 (diluted 1:500), anti-iNOS (diluted 1:1000); monoclonal mouse anibodies: and anti-NT (diluted 1:1000) and anti-rabbit IgG or anti-mouse as the secondary antibody (1:20 000). All antibodies used in this study were purchased from Santa Cruz Biotechnology (Santa Cruz, CA, USA). The immunoactive proteins were determined using an enhanced chemiluminescence (ECL) Western blotting detection kit (Amersham Biosciences, Piscataway, NJ, USA). The same membrane was stripped and β-actin (Sigma Aldrich) was used as an internal control. Western blotting analysis and protein bands quantification was performed as previously reported by our group [22,35].

### Cell apoptosis and necrosis

Apoptotic and necrotic cells were measured by Annexin V binding and propidium iodide uptake using apoptosis assay kit (BD Pharmingen, USA). hFOB 1.19 cells (5x10^6^) were seeded into 25-cm^2^ tissues culture flask. After two days cells were treated as indicated in section *Treatment*. Next, floating and adherent cells were collected and washed with PBS. Annexin V (5 μL) and propidium iodine (5 μL) were added to the cells, which were resuspended in binding buffer. The cells were gently shaken and incubated for 15 minutes at room temperature in the dark. Cells were further diluted with the buffer and analyzed using a BD FACSArray (BD Biosciences, USA). Ten thousand specific events were analyzed. A plots from the gated cells illustrated the populations corresponding to viable (Annexin V–PI–) cells, apoptotic (Annexin V+PI–) cells, apoptotic/necrotic (Annexin V+ PI+) cells and to dead (Annexin V- PI+) cells. Also, effect of Ag released from 30 and 60 μg/mL AgNPs (prepared as indicated in section *Characterization of AgNPs*) on apoptosis after 48 h of incubation (Annexin V+PI–) was evaluated (S4 Fig).

### Cell viability

Cell viability was measured by lactate dehydrogenase (LDH) assay (Promega, Poland). hFOB 1.19 cells were seeded in triplicate at a density of 10^4^ cells/100 μL of cell-culture medium into 96-well plate. The next day, hFOB 1.19 cells were exposure to AgNPs under SF conditions at the concentrations 1, 5, 15, 30, and 60 μg/mL for 18, 24, 48 hours as well as to Ag released from 30 and 60 AgNPs μg/mL (prepared as indicated in section *Characterization of AgNPs*) for 24 and 48 hours. LDH release into the surrounding medium was measured according to the manufacturer’s protocol. Absorbance values were corrected with blank NPs. LDH data were expressed as a percentage of the total LDH released from cells into the culture medium.

### RT-PCR

Real-time (RT)-PCR was used to measure mRNA levels of Bax in the absence or presence of L-NIL and iNOS. hFOB 1.19 cells were cultured in T-25 cm^2^ flasks in complete medium until 80–90% confluent. The cells were treated as indicated in section *Treatment*. Next, medium was aspirated, the cells were rinsed with phosphate-buffer saline and collected. DNA-free RNA was isolated using a RiboPure Kit (Ambion, Huntingdon, UK according to the supplier’s instruction and RNA in each sample was quantified using a NanoDrop ND-1000 spectrophotometer (Fisher Scientific, Ireland). Then, DNA-free RNA from samples were reverse-transcribed using a high-capacity cDNA reverse transcription kit (Applied Biosystems, UK). RT-PCR as well as quantitative analysis was conducted as previously reported by our group [22,29,36].

The sequences of the primers of iNOS (accession number: XM_034166), Bax (accession number: NM_004324) and Bcl-2 (accession number: NM_000633.2) were: 5'-ACAACAAATTCAGGTACGCTGTG-3',5' (junction exon 15/16) TCTGATCAATGTCATGAGCAAAGG-3' (exon 16) and 5'-TGGAGCTGCAGAGGATGATTG-3' (junction exon 4/5), 5'-GAAGTTGCCGTCAGAAAACATG-3' (exon 5), and forward primer (exon 2): 5′-GGTGCCACCTGTGGTCCA-3′, reverse primer (exon 3): 5′ACTTGTGGCCCAGATAGG-3′, respectively. The location of forward primers ensure detection only mRNA of selected gene. The PCR conditions were as follows: incubation for 10 min at 95°C, followed by 45 cycles of denaturation at 95°C for 10 sec, annealing at 60°C for 15 sec, and extension at 72°C for 20 sec. The expression of each gene was normalized against 18S rRNA (accession number: X03205) expression and expressed relative to the control sample using the formula 2−(ΔΔCt), in which ΔΔCt = (Ct mRNA–Ct 18S rRNA) sample–(Ct mRNA–Ct 18S rRNA) control.

### Nitric oxide measurement

The levels of NO in the presence or absence of AgNPs were measured following conversion of nitrate to nitrite using the Griess reagent (Stressgen colorimetric diagnostic kit). Briefly, the cells were seeded into 24-well plates at a concentration of 10^5^ cells/well. 24 hours after culturing, cells were incubated in the presence or absence of L-NIL as indicated in section *Treatments*. Nitrite concentrations were determined using a standard curve of sodium nitrate and nitrite levels in the cell culture supernatants. Media without cells in the presence of the of 30 or 60 μg/mL AgNPs was used as background control and their absorbance were subtracted from the reading of the samples.

### Nitrotyrosine measurement

The level of 3-nitrotyrosine, as an index of NO-derived reactive species [24,37] was detected by ELISA kit (Abcam, Poland). The hFOB 1.19 cells were seeded into 12-well plates at a concentration of 10^6^ cells/well. 24 hours after culturing, cells were incubated in the presence or absence of L-NIL as indicated in section *Treatments*. Briefly, cells were collected and rinsed twice with phosphate buffered saline. Cells pellet were solubilized in Extraction Buffer (provided by supplier), incubated on ice for 20 minutes and centrifuged at 16000 x g 4°C for 20 minutes. Nitrotyrosine levels were measured according to instruction provided by the supplier. The absorbance was measured at 450 nm and nitrotyrosine concentrations were determined using a standard curve of a nitrotyrosine labeled albumin. Absorbance values were also corrected with blank AgNPs. Results were expressed as nmol/mg protein.

### iNOS siRNA silencing

In brief, hFOB 1.19 cells were grown to 80% confluence in 6-well plates and transfected with iNOS siRNA using Lipofectamine 2000 (Invitrogen, Poland) in accordance with the protocol of the manufacturer. To control for possible non-specific effects of siRNA, Stealth RNAi™ siRNA negative controls were applied. To optimize the efficiency of transfection, different ratio mixtures of siRNA and Lipofectamine were investigated (data not shown) and 50 nM siRNA was selected. Optimal results were achieved at 24 hours. Effective knockdown of iNOS was confirmed by Western blot analysis. 24 hour after transfection, cells were exposed to 60 μg/mL AgNPs (the highest tested concentration) for 48 h and subjected to western blot, flow cytometer experiments as well as determination of NO and NT level as described above. Cells treated with SF culture medium without NPs were used as a control.

### Protein content

Protein-content was measured by the method of Bradford [38] using Coomassie Protein Assay Kit (Thermo Fisher Scientific, USA). Measurements were performed according to protocol provided by supplier.

### Statistical analysis

All data are presented as the mean ± standard error of 3–4 independent experiments. Statistical analysis was determined by one-way analysis of variance and Tukey’s post-hoc test, and *P*-values <0.05 were considered statistically significant. All statistical analyses were performed using GraphPad Prism software (GraphPad Software, Inc, La Jolla, CA, USA). Pearson correlation analysis was performed to study the relation between concentration of AgNPs and iNOS at protein and mRNA level.

## Results and Discussion

This study provides the evidence that 18 nm uncoated AgNPs enter the cell and that NO generated by iNOS is implicated in AgNPs-induced apoptosis and necrosis in human hFOB 1.19 cells, an established osteoblast model [39]. Importantly, preincubation of osteoblast cells with (L-NIL), a selective iNOS inhibitor, as well as silencing of the iNOS with siRNA significantly attenuated / protected AgNPs-induced apoptosis and necrosis confirming the involvement of iNOS in this process.

### Characterization of AgNPs

**Fig 1:**
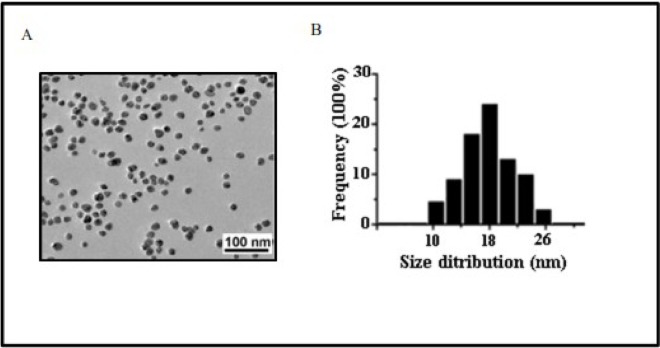
Characterization of AgNPs using transmission electron microscopy (TEM). (A) A representative microscopy image shows shape of AgNPs. (B) The histogram illustrates the range of particle size distribution with the mean size of 18.3 nm obtained from TEM measurements of 200 particles.

Physicochemical properties of NPs can determine pharmacological and toxicological outcomes of NPs-cell interactions [7,21,22]. It has been reported that significant deviations from nominal specifications are present in commercially supplied samples [21]. Therefore, first, we characterize the supplied AgNPs dispersed in ultrapure water or serum free culture medium, suitable for hFOB 1.19 cells using TEM and Zetasizer. During TEM analysis we have found that AgNPs had a regular, spherical shape and sizes ranging from 10 to 26 nm with a mean diameter of 18.3±2.6 nm, which is similar to size specified by manufacturer: 15 nm (Fig 1). To characterize AgNPs under the conditions of biological exposure the zeta potential and the polydispersity index were measured after dispersion of AgNPs (30 and 60 μg/mL) in serum free cell culture medium using Zetasizer. No significant differences in characterization between two used concentration of AgNPs was observed, therefore data obtained for 60 μg/mL AgNPs are shown, as representative. From the obtained data (presented in Table 1) it was evident that AgNPs in serum free media were negatively charged and monodispersed at concentrations applied in the study [40]. The soluble Ag present in SF medium after 24 and 48 h exposure to AgNPs at the highest working concentration of 60 μg/mL was detected to be less than 0.3 μg/mL, which correspondents to less than 0.5% release (Table 1). These results are in agreement with a study by Gliga et al who detected a small portion of released Ag from AgNPs in cell culture medium [34]. In accordance with other studies [41], AgNPs formed aggregates in SF culture medium measured by DLS method. The effective diameter (hydrodynamic size) of AgNPs increased in both concentration- and time-dependent manner (Table 1), presumably because of the high concentration of ionic salts in the medium. It was also confirmed that the presence of L-NIL (500 μM) or Nec-1 (60 μM) in the analyzed samples of AgNPs did not affect investigated parameters (data not shown)

**Table 1:** The summary characteristics of AgNPs.

Characterization	Method	Results
**Size (nm)**	TEM	18.3±2.6
**Shape**	TEM	Spherical
**Polydispersity index (PDI)**	DLS	0.121±0.038
**Zeta potential (mV)**	DLS	-29.1±2.4
*Soluble Ag released (%)	AAS	< 0.5
**Hydrodynamic diameter (nm)**	DLS	
**30 μg/mL**		• **,,0” h:** 24 • **24 h:** 64±7 • **48 h:** 139±6
**60 μg/mL**		• **,,0” h:** 31±9 • **24 h:** 88±10 • **48 h:** 171±8

The polydisperity index (PDI) and zeta potential value were obtained from the dynamic light scattering (DLS) measurements using zetasizer. The DLS data were obtained for AgNPs dispersed in SF culture medium at the concentration of 60 μg/mL; the measurements were performed four times at room temperature.

*The amount of released Ag in SF cell medium after 24 and 48 h at 37°C, 5% CO_2_ and centrifugation was quantified by AAS and expressed as percentage from the total added AgNPs. Changes in the hydrodynamic diameter of 30 and 60 μg/mL AgNPs is presented after, 0”, 24 and 48 hours in SF cell medium at 37°C, 5% CO_2_. Results are presented as mean ± standard deviation of 4 replicates.

### Uptake of AgNPs by hFOB 1.19 cells

**Fig 2:**
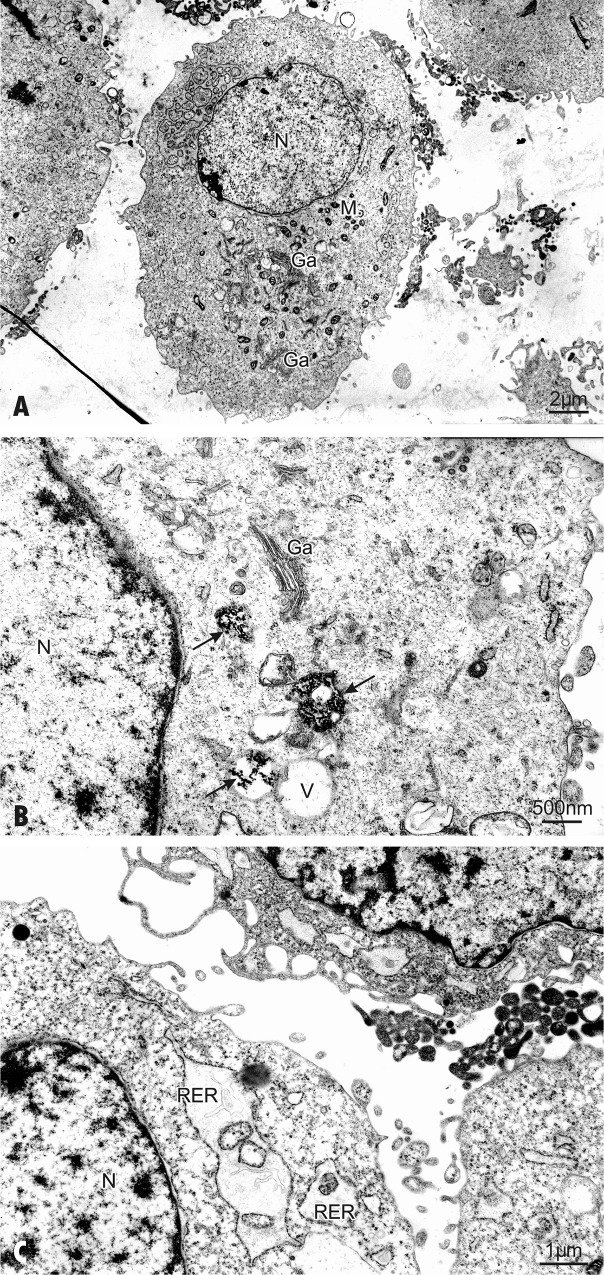
The TEM examination of hFOB 1.19. (A) The ultrastructural features of a control cell; mitochondria (M), nucleus (N), Golgi complex (Ga); (B-C) Cells exposed to AgNPs 30 μg/mL for 24 h. cell. (B) AgNPs are found within the cell (arrows). Micrographs show vacuoles (V) with randomly oriented AgNPs of quite regular morphology (arrows). The well-developed Golgi complex (Ga) is located in the cytoplasm in perinuclear areas. (C) Cells exposed to AgNPs 30 μg/mL for 24 h show swelling of the endoplasmic reticulum. Ribosomes may have dissociated from the endoplasmic reticulum (RER) and the cytoplasm is filled with free ribosomes. The nucleus (N) presents a normal aspect.

We used transmission electron microscopy (TEM) for ultrastructural examination of AgNPs-exposed osteoblast cells. The process of fixing, dehydrating, staining, resin embedding, and ultramicrotome sectioning allowed us to visualize uptake and intracellular locations of AgNPs. The electron micrograph (Fig 2B) shows osmiophilic granular aggregates of AgNPs localized frequently in cellular vacuoles. Single electron dense particles were spherical in shape and the average size of 18 nm.

In keeping with our findings, Sengstock et al showed that 80 nm AgNPs were taken up by human mesenchymal stem cells and after 24 h of treatment were visible in the endo-lysosomal cell areas, but not inside the nucleus [42]. The translocation of nanoparticles to the nucleus appears to be size-dependent. Indeed, metal (Au) NPs with a size of 5 nm were found in nuclei of human fibroblast cell line, whereas nanoparticles with size 30 nm or larger were observed in the cytoplasm [43]. However, other study demonstrated that exposure of osteoblast cells to AgNPs with a size 5 nm resulted in their localization inside the lysosomes but not inside the nucleus [18]. On the other hand, nuclear presence has been detected for AgNPs with size 30 nm in HaCaT cells and >50 nm NPs were detected in human mesenchymal stem cells [44,45]. Also, He et al found that 24 h exposure to AgNPs (30 nm) resulted in their distribution in the cytoplasm, the nucleus and different sized vesicles in human bone marrow-derived mesenchymal stem cells [10]. We have previously found that treatment of human gingival fibroblast cells with 2 AgNPs nm caused their uptake and localization mainly in the mitochondria [22]. However, when human osteoarthritic chondrocytes were incubated for 24 h in the presence of 65 nm AgNPs (250 μM) the intracellular uptake of these NPs could not be detected by TEM [15]. Finally, Park et al described that when RAW264.7 mouse macrophage-like cells were exposed to Ag-NPs, the NPs were found in the cytosol of activated but not damaged cells [46]. The discrepancy in these findings indicates that cellular uptake of AgNPs is very complex and probably depends on many factors, such as size, functionalization, zeta potential, concentration, time of incubation as well as on a type of cells.

### Cytotoxicity of AgNPs in hFOB 1.19 cells

**Fig 3:**
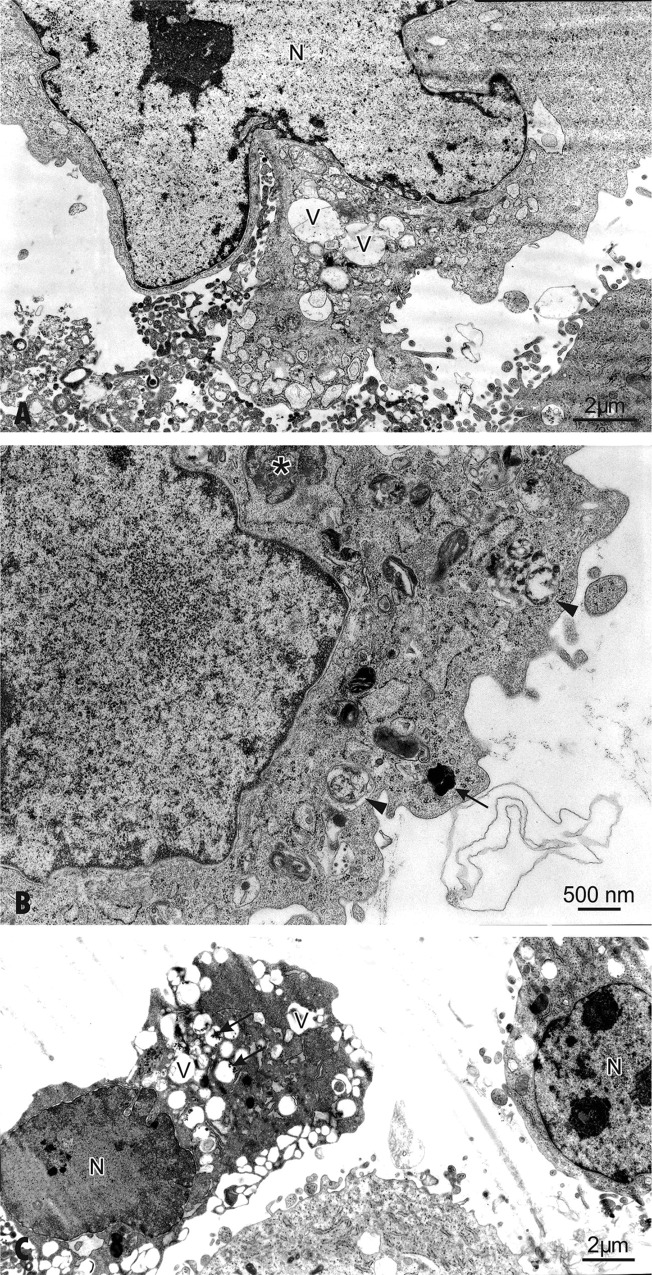
The ultrastructural alterations of hFOB 1.19 cells after exposure to 30 or 60 μg/mL AgNPs for 48 h. (A) Cells show ultrastructural changes and formation of multiple blebs. Enlarged nucleus (N), pushing the cytoplasm towards the periphery. (B) The cell exposed to 30 μg/mL AgNPs for 48 h contains double-membraned autophagic vacuoles—autophagolysosomes with engulfed organelles displaying degenerative changes (arrowheads). Autophagosome (asterisk) and lysosome (arrow) are indicated. Note condensed mitochondria and disorganization of the inner membrane system. (C) Apoptosis in cells exposed to 60 μg/mL AgNPs for 48 h. A decrease in cell volume and chromatin condensation show induction of apoptosis in the osteoblast on a right side of figure. Note a loss of microvilli and formation protrusions from the surface of plasma membrane—known as blebs. The autolytic vacuoles are visible in the cytoplasm. Features of necrotic lysis, such as disintegration of cytoplasmic membrane, electron-lucent nuclear chromatin, heavily vacuolized cytoplasm (arrows) and lack of organelles are prominent.

Gliga et al have shown that the intracellular uptake of AgNPs is responsible for the nanoparticle cytotoxicity [34]. We have also observed that the uptake of AgNPs by osteoblasts is associated with alterations in cell ultrastructure and signs of injury (Figs 2C and 3A–3C). Indeed, osteoblasts exposed to AgNPs at concentration of 30 μg/mL showed the first manifestation of cell injury, such as: swelling of the endoplasmic reticulum [47] (Fig 2C). On closer ultrastructural examination AgNPs-treated cells showed morphological features characteristic for apoptosis (Fig 3C) including clumping and margination of condensed heterochromatin at the nuclear periphery, decreasing cell volume, increasing nuclear to cytoplasm ratio, apoptotic body formation, loss of microvilli and plasma membrane blebbing in individual cells. This is consistent with the study conducted by Pascaralli et al who showed an increased number of osteoarthritic chondrocytes with evident signs of apoptosis, after treatment with AgNPs at concentrations (160 and 250 μM) [15]. In addition to hallmarks of apoptosis we have also found, ultrastructure changes indicative of necrosis following incubation of hFOB 1.19 with 60 μg/mL AgNPs (Fig 3C).

Interestingly, the ultrastructural examination showed also signs of autophagy (Fig 3B). This is not surprising given that autophagy is generally thought of as a survival mechanism that regulates the extent of apoptosis and necrosis [48]. During autophagy, parts of the cytoplasm are sequestered within characteristic double-membraned autophagic vacuoles, called autophagosomes and are finally delivered to lysosomes for degradation. Interestingly, the development of double-membrane autophagosomes was observed in various type of AgNPs-treated cells, e.g. mouse embryonic fibroblast cells, male somatic Leydig and Sertoli cells and human liver derived hepatoma cells [49–51]. It has to be emphasized that, the cross talk between autophagy and apoptosis as well as autophagy, apoptosis and NPs is very complex and still not completely understood. On one hand, deregulated autophagy after NPs treatment may lead to increased cell death, either independently or in conjunction with apoptosis or necrosis [48,51–53]. On the other hand, autophagy is also a physiological process maintaining cellular homeostasis and may appear independent of its role in cell death and promote cell survival [53,54]. Interestingly, exposure of adipogenic and osteogenic cells to 10 and 20 nm AgNPs resulted in cellular uptake and decreased cell viability in the absence of significant ultrastructural alterations [16].

### What molecular mechanisms may be responsible for cytotoxic effects of AgNPs in hFOB 1.19 cells?

**Fig 4:**
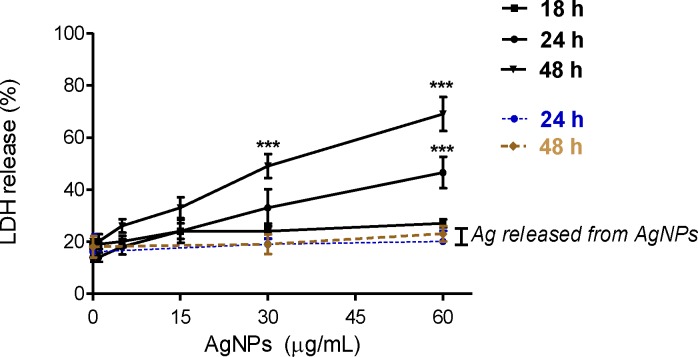
Evaluation of cytotoxicity of AgNPs and Ag released in cell medium from 30 and 60 μg/mL AgNPs on hFOB 1.19 cells. Results are expressed as % LDH release by cells into the culture medium and presented as mean ± standard deviation of 3 independent experiment. ***p<0.001 exposed cells v/s control.

AgNPs may cause cell death both *in vitro* and *in vivo*. Indeed, AgNPs enhanced apoptosis and upregulation of the p53-related pro-apoptotic genes Bax, Noxa, p21 in the liver of adult zebrafish [55]. The changes in mRNA regulation were caused by AgNPs-induced free radicals. Similarly, other study suggested that AgNPs induced apoptosis through increasing intracellular ROS production in cultured cerebral cortical neurons [56]. Ahamed et al demonstrated that activities of caspase-3 and caspase-9, markers of apoptosis were significantly higher in *Drosophila melanogaster* exposed to AgNPs [57]. Necrosis induction by AgNPs exposure in cell culture was also documented. Treatment of epidermoid larynx carcinoma cells with 31 nm AgNPs resulted in a significant elevation of LDH activities in the cell culture medium [58]. Finally, Gliga et al observed that 10 nm AgNPs caused membrane damage of human lung cells [34]. The cytotoxicity of AgNPs in osteoblasts appears to be dependent on the size of nanoparticles. For example, Kim et al ascertained that AgNPs enhanced apoptosis in mouse osteoblastic cells (MC3T3-E1) in nanoparticle size-dependent manner [59]. AgNPs < 10 nm induced apoptosis, but not 50 and 100 nm. The coating of NPs can also influence their cytotoxicity. However, the effect is not fully understood. For example, Suresh et al demonstrated that poly(diallyldimethylammonium)-coated AgNPs, biogenic-Ag and oleate-AgNPs were more toxic to macrophage and lung epithelial cells than uncoated AgNPs [60]. Gliga et al showed that cytotoxicity of 10 nm AgNPs in human lung cells was independent of surface coating [34]. Some studies found that toxicity of AgNPs was reduced due to the coatings, which limited direct contact of particle surface with cellular components [61] or have protective effect on the release of Ag ions [62]. It has been demonstrated that the toxicity of AgNPs is associated with the release of Ag ions [63,64]. However, not only Ag dissolution is the factor contributing to uncoated AgNPs-induced cytotoxicity [62]. Indeed, in our experiment no LDH leakage was detected when osteoblast cells were exposure to Ag released from AgNPs (Fig 4), indicating that the toxic effects observed after 24 and 48 h were not due to dissolved Ag in cell culture medium. Apoptosis was also not affected by released Ag (S4 Fig). Similarly, Stoehr et al demonstrated that Ag wires-induced loss of human alveolar epithelial cells viability was not due to released Ag ions in cell culture medium [65]. Also, Kim et al suggested that AgNPs-induced cytotoxicity is independent of the toxicity of Ag ions [66].

**Fig 5:**
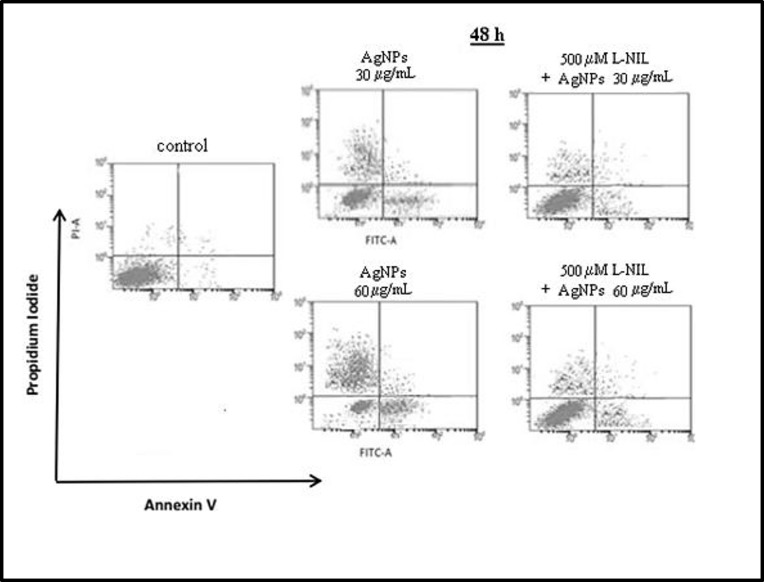
Pretreatment of hFOB 1.19 with L-NIL attenuated AgNPs-induced cell death. **A representative dot plot of flow cytometry.** A representative dot plot of flow cytometry. AgNPs induced apoptosis and necrosis in hFOB 1.19 cells an effect attenuated by L-NIL. Viable cells are shown in the lower left field (low Annexin V and PI staining; AV- PI-). The lower right field (AV+ PI-) represents the apoptotic cells, and the higher right field (AV+ PI+) indicates late apoptotic/necrotic cells. The higher left field (AV- PI+) shows the dead cells. L-NIL significantly attenuated number of apoptotic and dead cells.

**Fig 6:**
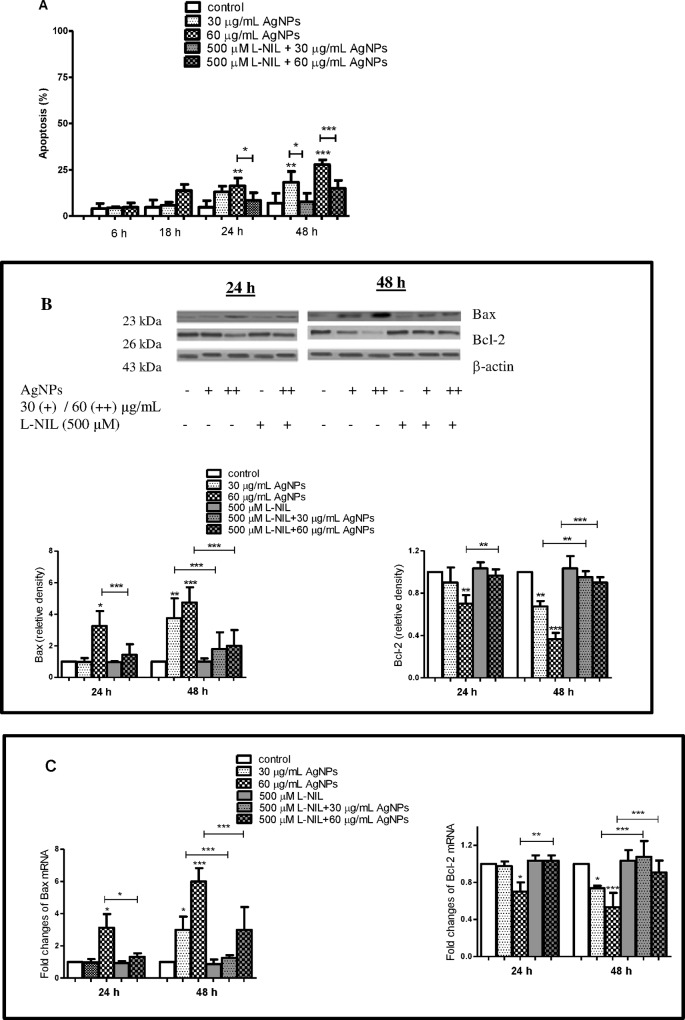
AgNPs-induced apoptosis in hFOB 1.19 cells and protective effect of L-NIL. (A) AgNPs-induced apoptosis, (B) Bax, Bcl-2 protein and (C) Bax, Bcl-2 mRNA levels. L-NIL significantly attenuated: (A) number of apoptotic cells, (B) Bax protein, (C) mRNA level and significantly increased: (B) Bcl-2 protein, (C) mRNA level. Data are expressed as means ± SD of 3 independent experiments. *p<0.05; **p<0.01; ***p<0.001 exposed cells v/s control or as indicated.

**Fig 7:**
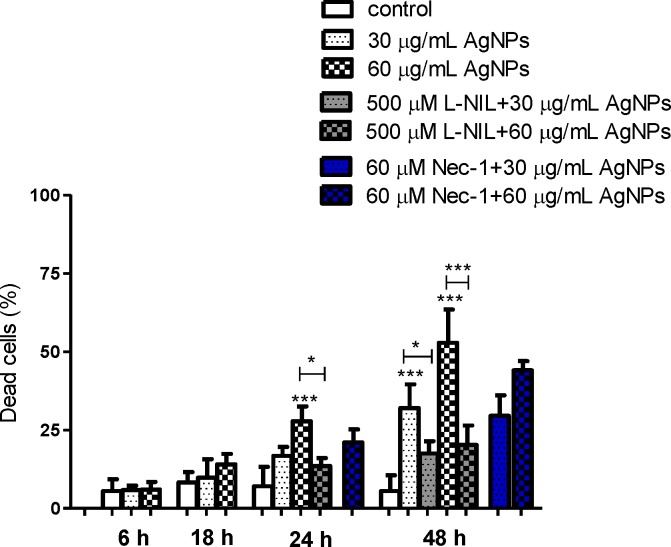
AgNPs-induced necrosis in hFOB 1.19 cells and protective effect of L-NIL. Nec-1 failed to protect cells from AgNPs-induced cell death. Data are expressed as means ± SD of 3 independent experiments. *p<0.05; ***p<0.001 exposed cells v/s control or as indicated.

In our study, we have found that uncoated AgNPs with size around 18 nm enhanced significantly both a number of apoptotic (Annexin V+ PI-) (Figs 5 and 6A) and dead cells (Annexin V- PI+) (Figs 5 and 7) in a concentration- and time-dependent manner.

In these experiments apoptosis was significantly increased from 4.7% (control) to 16.2% following incubation with 60 μg/mL AgNPs for 24 h and from 7% to 18.2% and 27.7% following incubation with 30 and 60 μg/mL AgNPs for 48 h, respectively. This increased number of apoptotic cells was associated with a concentration- and time-dependent increase in proapoptotic Bax and decrease in anti-apoptotic Bcl-2 protein and mRNA levels (Fig 6B and 6D). The levels of Bax protein increased significantly 3.2, 3.8, 4.8-fold following incubation with 60 μg/mL AgNPs for 24 h and with 30 and 60 AgNPs μg/mL for 48 h, respectively compared to the control. The levels of Bax mRNA in osteoblast cells increased significantly 3.1, 2.9, 6-fold following incubation with 60 μg/mL AgNPs for 24 h and with 30 and 60 AgNPs μg/mL for 48 h, respectively compared to the control. Moreover, the levels of Bcl-2 protein decreased significantly (p<0.01 or p<0.01, p<0.001, n = 3) following incubation with 60 μg/mL AgNPs for 24 h and with 30 and 60 AgNPs μg/mL for 48 h, respectively compared to the control. The levels of Bcl-2 mRNA in osteoblast cells decreased significantly (p<0.05 or p<0.001, n = 3) following incubation with 60 μg/mL AgNPs for 24 h and with 30 and 60 AgNPs μg/mL for 48 h, respectively compared to the control. The presence of the late apoptotic/necrotic cell (Annexin V+ PI+) was very little, not statistically significant. The percentage of dead cells (Annexin V- PI+) osteoblasts increased significantly from 7% (control) to 27.7% following incubation with 60 μg/mL AgNPs and from 6% to 32% and 52.8% following incubation with 30 and 60 μg/mL AgNPs for 48 h, respectively. This results together with ultrastructural observation by TEM and a significant increase of LDH release suggested that necrosis as well as necroptosis may be involved in AgNPs-induced osteoblast death. Indeed, a novel form of cell death, termed necroptosis, shows morphological features similar to necrosis [67]. However, necroptosis is strictly regulated forms of cell death and can be specifically inhibited by Nec-1 [67]. In our study, percent of dead cells (staining with PI) did not significantly reduce when they were pretreated with Nec-1 (Fig 7). These data indicated that necrosis, but not necroptosis is associated with AgNPs-induced cells death in hFOB 1.19 cells. Nonetheless, our results support the notion that nanoparticles, could cause cell death through a complex mechanism, including apoptosis, necrosis, apoptosis-like, and necrosis-like process [68] and that several types of cell death may be activated simultaneously within cells [53].

### AgNPs induce expression of iNOS and generation of NO and nitrotyrosine formation in hFOB 1.19 cells

**Fig 8:**
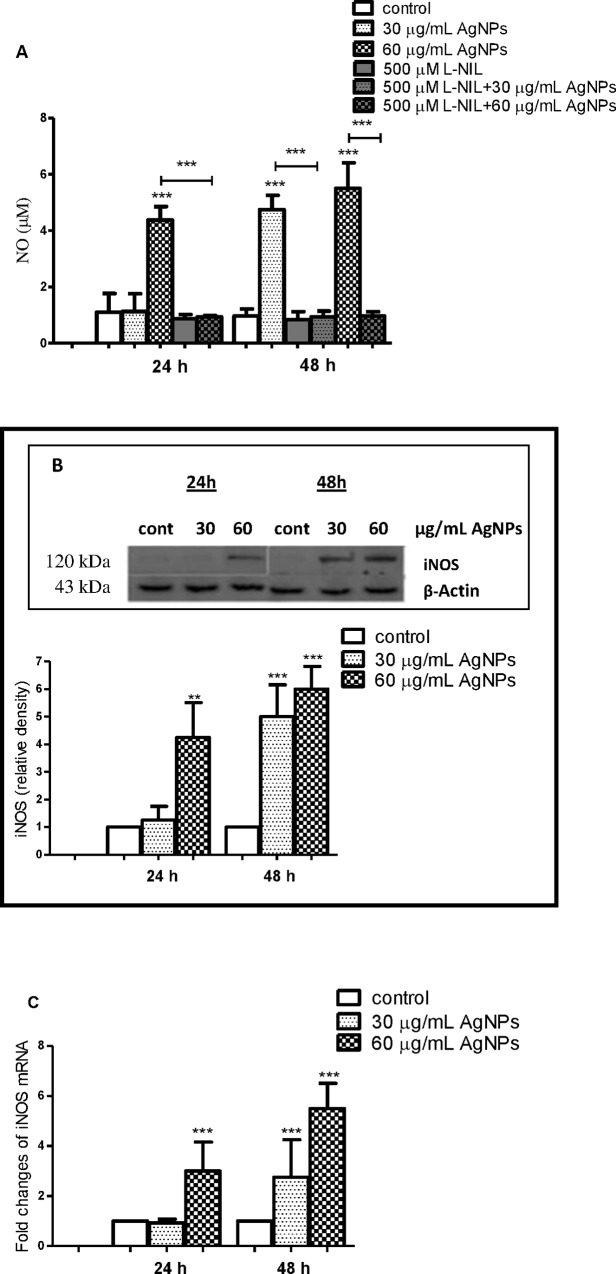
AgNPs-induced expression of iNOS in hFOB 1.19 cells. (A) AgNPs increased generation of NO and its inhibition by L-NIL. (B) Immunoblot of iNOS and (C) mRNA levels. Data are expressed as means ± SD of 4 independent experiments. **p<0.01; ***p<0.001 exposed cells v/s control (con) or as indicated.

It has been shown that NO exerts dual role in osteoblast activities. The constitutive NO plays an important role in regulation of osteoblast proliferation and differentiation [27,28,69]. On the other hand, overproduction of NO in response to iNOS expression results in osteoblast injury and may contribute to the pathogenesis of bone diseases [25,32,70,71]. Therefore, we have decided to study the impact of AgNPs on the expression of iNOS and generation of NO in hFOB 1.19 cells. We found that AgNPs enhanced production of NO in osteoblast cells in a concentration- and time-dependent manner. As shown on Fig 8A incubation of cells for 24 h with 60 μg/mL and for 48 h with 30 and 60 μg/mL AgNPs resulted in a significant increase in NO levels (p<0.001, n = 4).

Importantly, the addition of 500 μM L-NIL, a selective inhibitor of iNOS [70] prevented AgNPs-induced increase in NO levels indicating that iNOS is responsible for this increase (Fig 8A). As expected, AgNPs increased the expression of iNOS, both at protein (p<0.001, n = 4) and mRNA level (p<0.001, n = 4) in hFOB 1.19 cells (Fig 8B and 8C) in a concentration- and time-dependent manner. Furthermore, a significant positive correlation between AgNPs concentration and iNOS protein and mRNA levels was found (Table 2).

**Table 2:** Pearson’s correlation coefficient between AgNPs and iNOS.

Parameters	n	r	p
**AgNPs/iNOS protein level**	18	0.837	<0.001
**AgNPs/ iNOS mRNA level**	18	0.721	<0.001

n-number of observation per parameter; r-correlation coefficient

**Fig 9:**
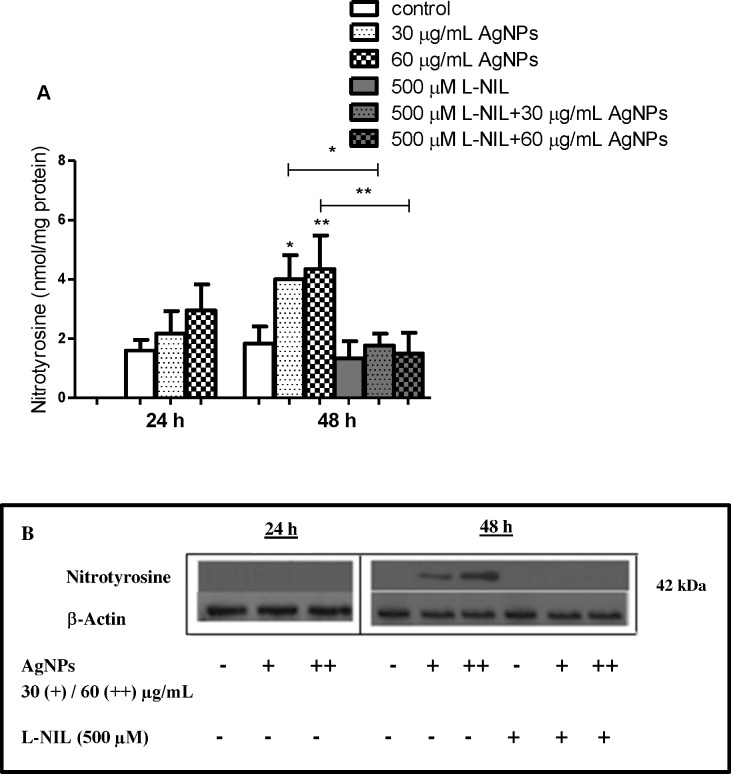
AgNPs-induced generation of nitrotyrosine in hFOB 1.19 and its inhibition by L-NIL. (A) A bar graph showing data as measured by ELISA; (B) Representative immunoblots. Data are expressed as means ± SD of 3 independent experiments. *p<0.05; **p<0.01 exposed cells v/s control or as indicated.

Interestingly, Pascarellia et al during immunocytochemical study of osteoarthritic chondrocytes observed that 160 and 250 μM AgNPs with size 65 nm significantly increased percentage of cells with an intense iNOS signal inside the cytoplasm compared to the controls [15]. Ramírez-Lee et al reported similar findings in airway smooth muscle cells [72]. Thus, increased expression of iNOS appears to be a common denominator in AgNPs-cell interactions leading to generation of high levels of NO. It has to be noticed that increased generation of NO may also result in the synthesis of powerful oxidants, such as ONOO^-^ and NO_2_ [24, 73]. For example, silica nanoparticles induce excessive generation of NO in vascular endothelial cells leading to ONOO^−^ production and endothelial dysfunction [21]. ONOO^−^ is involved, at least partially, in NO-induced deleterious effects on human osteoblast metabolism [74]. Peroxynitrite-induced protein nitration may result in formation of NT [24,73,74]. Indeed, we found that an increase of NO production in hFOB 1.19 cells exposed to AgNPs for 48 h led to increased generation of NT (p<0.05, p<0.01, respectively; n = 4) as detected by ELISA and Western blotting. This effect was reversed by preincubation with 500 μM L-NIL (Fig 9A and 9B).

### iNOS expression is implicated in AgNPs-induced hFOB 1.19 cell death

It has been demonstrated that NO directly or following conversion to reactive nitrogen species may activate biological signaling to cause cells apoptosis [71,73,74]. A number of studies have explored the effects of increased generation of NO on osteoblast apoptosis. Chen et al demonstrated that NO released from NO donor induced osteoblast apoptosis and this was associated with reduced anti-apoptotic Bcl-2 protein [26]. Also, it was suggested that NO causes apoptosis in osteoblasts by increasing the synthesis of Bax protein *de novo* [75]. Damoulis and Hauschka found that NO is responsible for decreased mouse osteoblast (MC3T3-E1) viability [76]. Exposure of osteoblasts to sodium nitroprusside (SNP), a donor of NO, led to decreased osteoblast viability [77]. Therefore, we hypothesized that the expression of iNOS and increased generation of NO are implicated in AgNPs-induced apoptosis and necrosis in human osteoblasts. To explore our hypothesis, we measured AgNPs-induced apoptosis and necrosis in the presence or absence of L-NIL. Importantly, we found that pre-incubation of hFOB 1.19 cells with L-NIL significantly attenuated (60 μg/mL for 24 h and 30, 60 μg/mL for 48 h) AgNPs-induced apoptosis by 48, 49, 44%, respectively and necrosis by 52, 46, 61%, respectively (Fig 5, Fig 6A and Fig 7). The reduction of a number of apoptotic cells was accompanied by decreased expression of Bax mRNA (p<0.05 or p<0.001, n = 3) and protein levels (p<0.001, n = 3), which had been elevated following exposure to AgNPs (Fig 6B and 6C). Also, a significant increase of Bcl-2 at protein and mRNA level (p<0.01 or p<0.001, n = 3) was found (Fig 6B and 6C).

**Fig 10:**
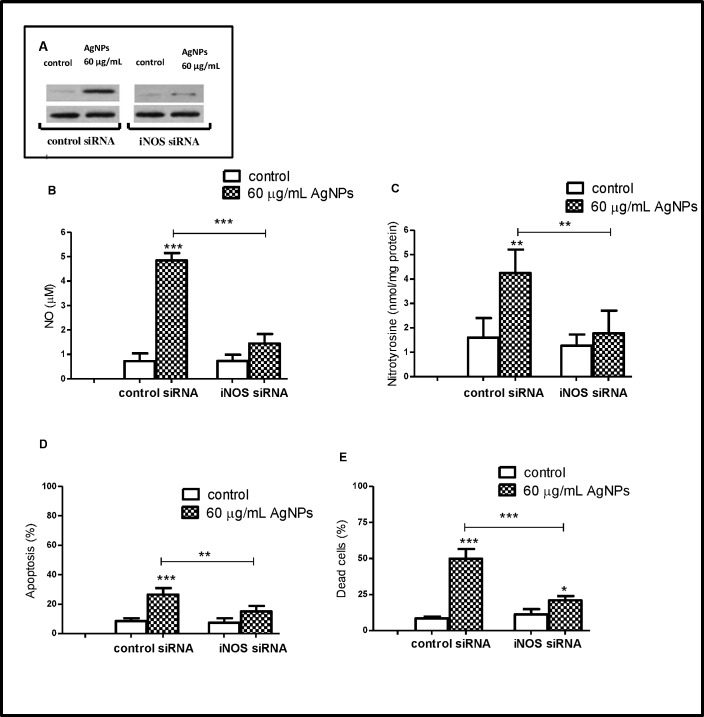
Transfection of hFOB 1.19 with iNOS siRNA prevents AgNPs-induced cell death. The hFOB 1.19 cells were transfected with either 50 nM iNOS or control siRNA for 24 h, then cells were exposed for 48 h to 60 μg/mL AgNPs (the highest working concentration). (A) A representative immunoblot demonstrates efficient of transfection of iNOS siRNA. iNOS siRNA prevents AgNPs-induced increase of (B) NO, (C) NT, (D) apoptosis level and attenuated AgNPs-induced (E) cell death. Data are expressed as means ± SD of 3 independent experiments. **p<0.01; ***p<0.001 exposed cells v/s control or as indicated.

To further investigate the link between iNOS and AgNPs-induced cell death, osteoblast cells were transfected with iNOS siRNA to silence iNOS expression. We found that in these cells, 60 μg/mL AgNPs failed to increase NO production, NT formation and apoptosis. Also, we have observed that iNOS siRNA significantly reduced the percent of AgNPs-induced dead cells (Annexin V- PI+) (Fig 10A–10E). These results confirmed the view that AgNPs induced apoptosis and necrosis in human osteoblasts by the iNOS pathway.

## Conclusion

We have found that the exposure of human osteoblasts *in vitro* to AgNPs with the average size of 18 nm results in nanoparticle uptake and changes in cell ultrastructure leading to apoptosis and necrosis. The cell death was associated with increased level of iNOS mRNA, iNOS protein and generation of increased amounts of NO. The detrimental effects of AgNPs could be attenuated by selective inhibition of iNOS activity using L-NIL or by silencing of the iNOS with siRNA. Thus, caution should be exercised when using AgNPs in prosthetic devices to prevent the nanoparticles toxicity. We also propose that selective inhibition of iNOS may be a novel strategy to increase the safety of AgNPs-containing prosthetic devices.

## Supporting Information

S1 Fig **AgNPs-induced apoptosis (A) and dead cells (B) in hFOB 1.19 cells after 48 h incubation.** Data are expressed as means ± SD of 3 independent experiments. **p<0.01; ***p<0.001 AgNPs-treated cells v/s control.
(TIF)

S2 Fig Pretreatment with L-NIL attenuated AgNPs-induced apoptosis in hFOB 1.19 cell after 48 h incubation. Depending on L-NIL concentration (100, 500, 1000 μM) reduction of AgNPs (60 μg/mL)-induced apoptosis in osteoblast cells. Data are expressed as means ± SD of 4 independent experiments. ***p<0.001 AgNPs-exposed cells v/s AgNPs-exposed cells in the presence of L-NIL.
(TIF)

S3 Fig Evaluation of Nec-1 on LDH release in hFOB 1.19 cells. Nec-1 at concentration 10–60 μM did not affect cells viability, measured by LDH release into the culture medium. Thus, 60 μM Nec-1 was selected for subsequent experiments. Data are expressed as means ± SD of 3 independent experiments. *p<0.05; **p<0.01 Nec-1-exposed cells v/s control.
(TIF)

S4 Fig Ag released in cell medium from 30 and 60 μg/mL AgNPs did not affect apoptosis in hFOB 1.19 cells after 48 h of incubation. Results are presented as mean ± standard deviation of 3 independent experiment.
(TIF)
