# Supplementary figures and images for: Molecular Mechanism of Silver Nanoparticles-Induced Human Osteoblast Cell Death: Protective Effect of Inducible Nitric Oxide Synthase Inhibitor
Source: PLoS One. 2016 Oct 7;11(10):e0164137. doi: 10.1371/journal.pone.0164137 (PMC5055295; doi:10.1371/journal.pone.0164137)

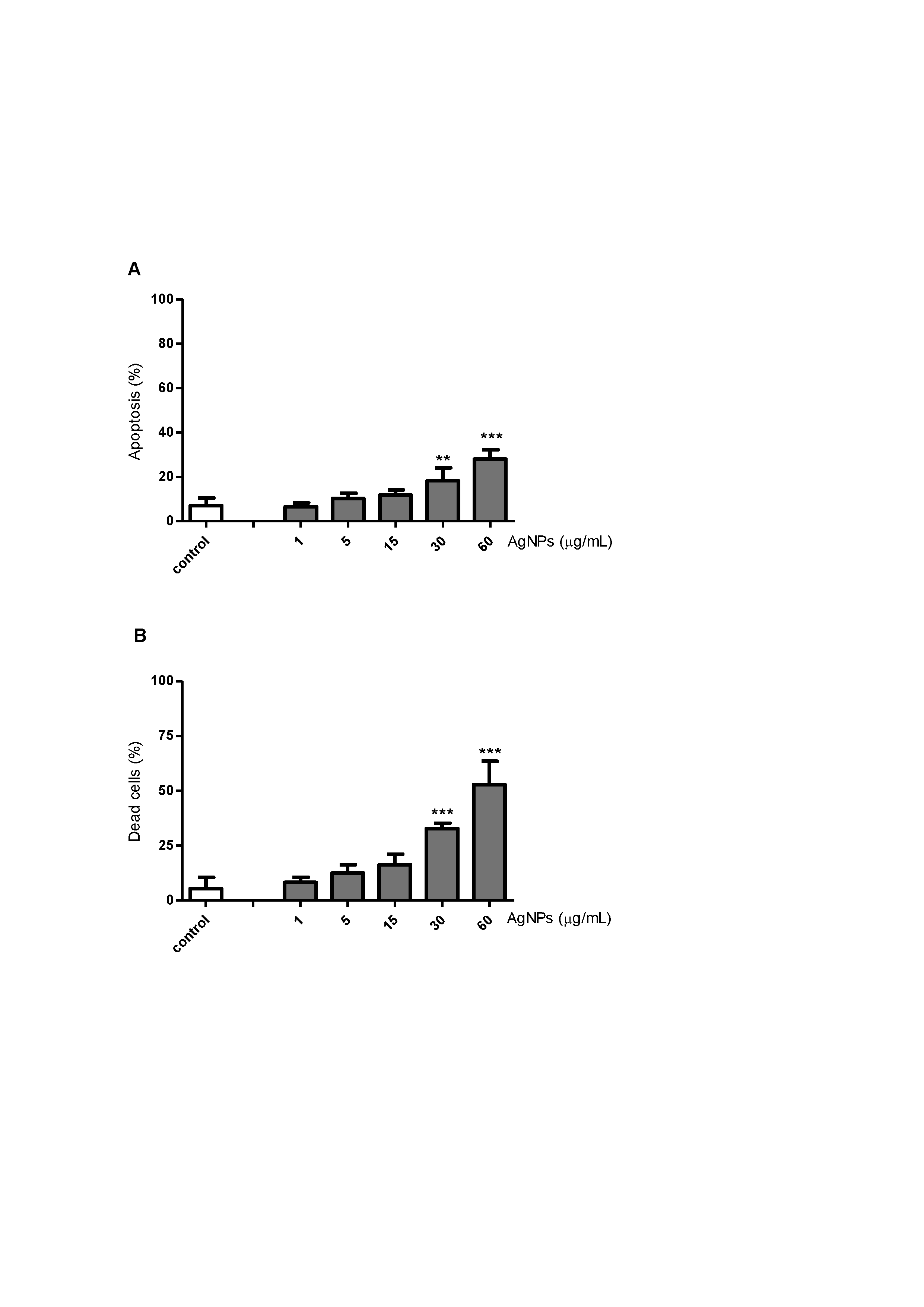

Supplement: S1 Fig — AgNPs-induced apoptosis (A) and dead cells (B) in hFOB 1.19 cells after 48 h incubation. Data are expressed as means ± SD of 3 independent experiments. **p<0.01; ***p<0.001 AgNPs-treated cells v/s control. (TIF) [file pone.0164137.s001.TIF]

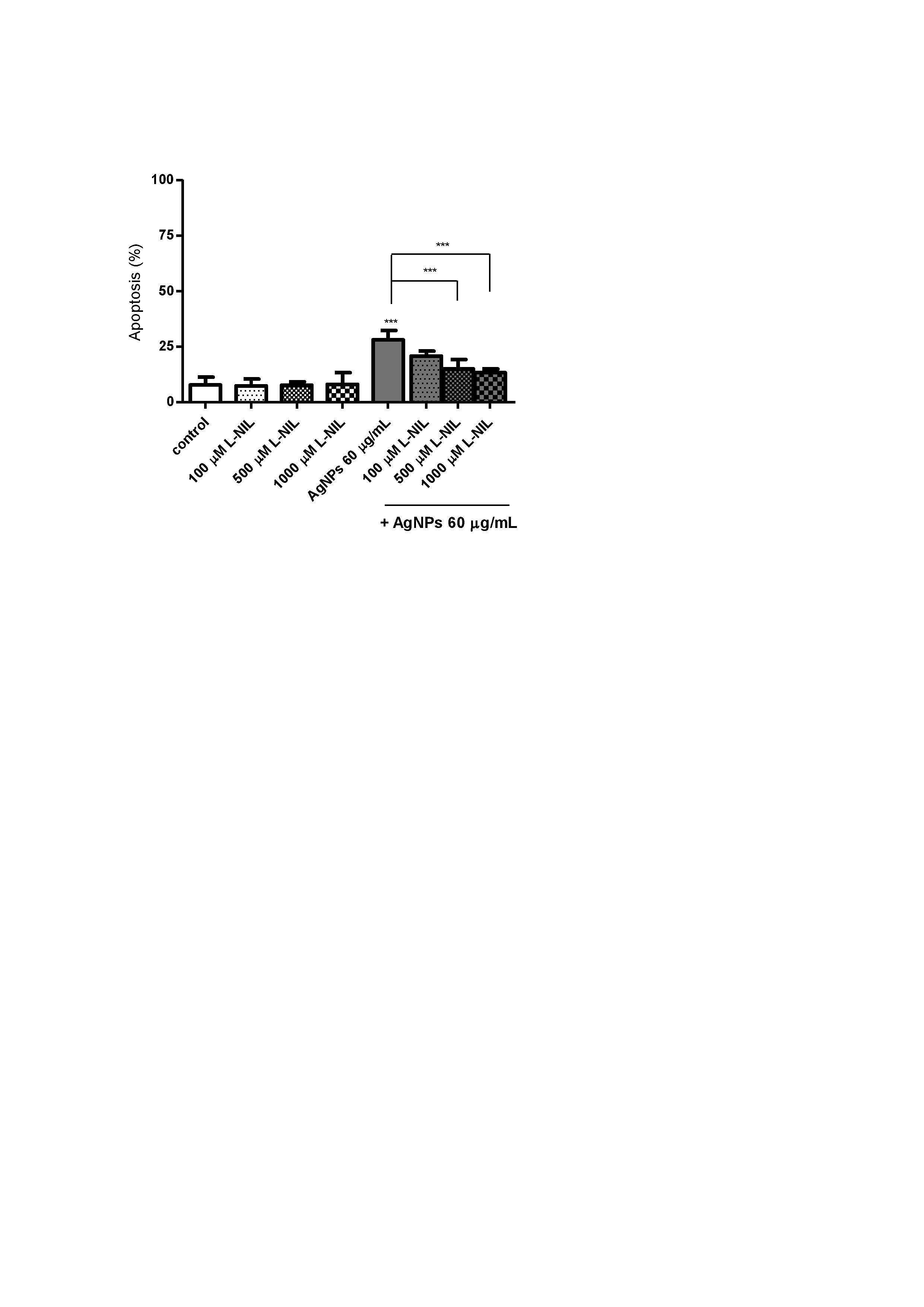

Supplement: S2 Fig — Depending on L-NIL concentration (100, 500, 1000 μM) reduction of AgNPs (60 μg/mL)-induced apoptosis in osteoblast cells. Data are expressed as means ± SD of 4 independent experiments. ***p<0.001 AgNPs-exposed cells v/s AgNPs-exposed cells in the presence of L-NIL. (TIF) [file pone.0164137.s002.TIF]

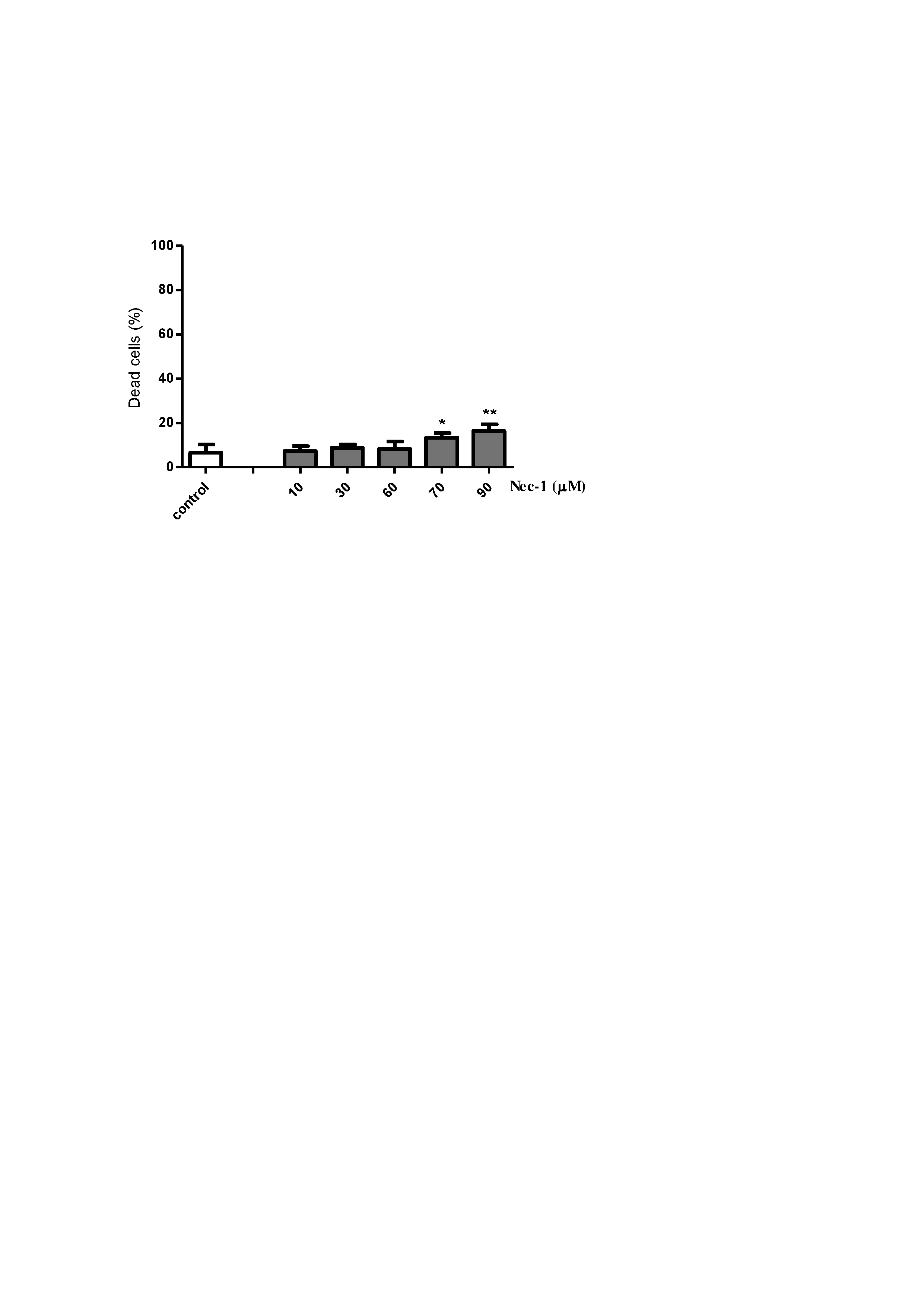

Supplement: S3 Fig — Nec-1 at concentration 10–60 μM did not affect cells viability, measured by LDH release into the culture medium. Thus, 60 μM Nec-1 was selected for subsequent experiments. Data are expressed as means ± SD of 3 independent experiments. *p<0.05; **p<0.01 Nec-1-exposed cells v/s control. (TIF) [file pone.0164137.s003.TIF]

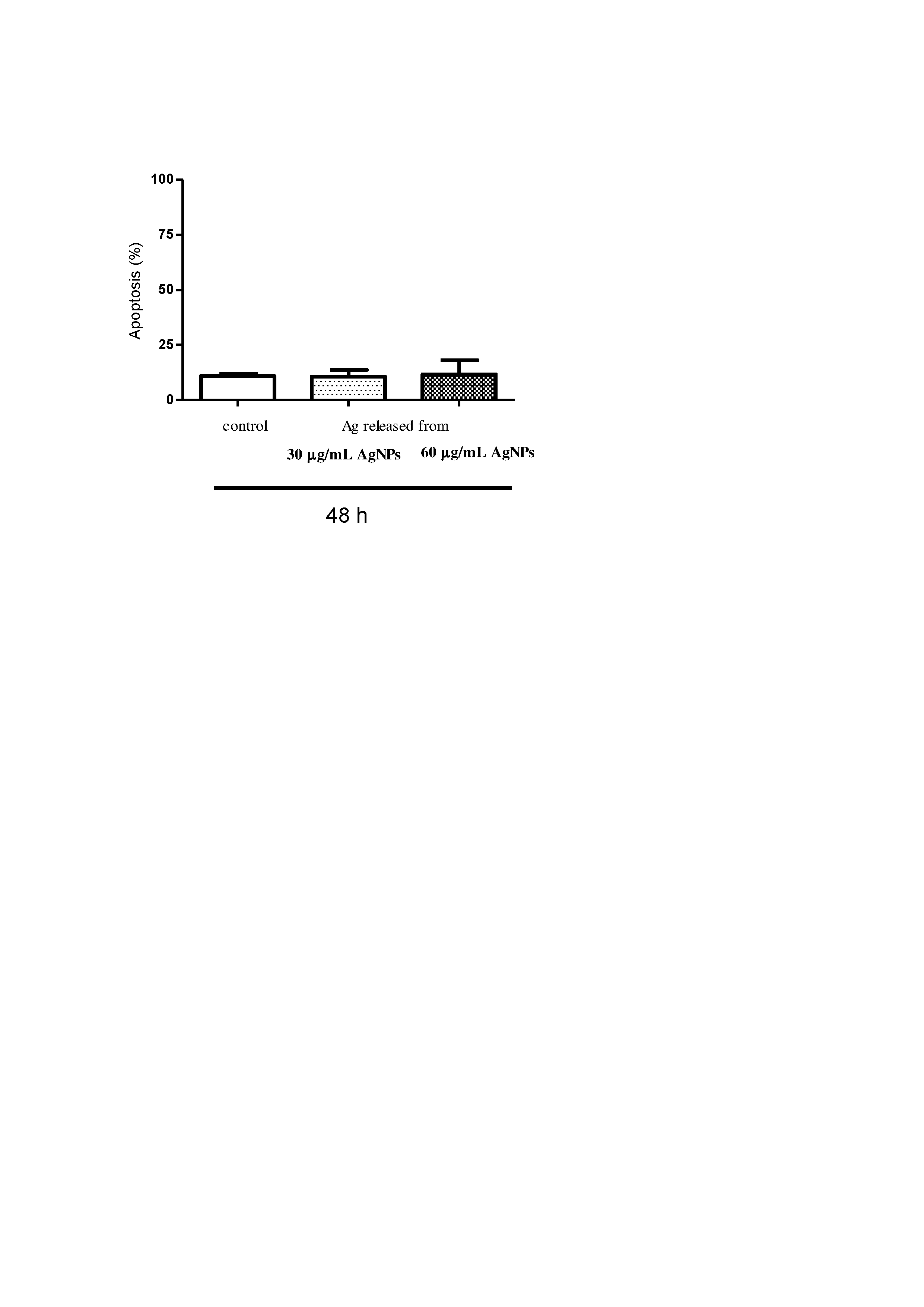

Supplement: S4 Fig — Results are presented as mean ± standard deviation of 3 independent experiment. (TIF) [file pone.0164137.s004.TIF]
